# l-carnitine and l-acetylcarnitine supplementation for idiopathic male infertility

**DOI:** 10.1530/RAF-20-0037

**Published:** 2020-12-23

**Authors:** Shen Chuen Khaw, Zhen Zhe Wong, Richard Anderson, Sarah Martins da Silva

**Affiliations:** 1 NHS Tayside, Ninewells Hospital, Dundee, UK; 2 International Medical University (IMU), Bukit Jalil, Kuala Lumpur, Federal Territory of Kuala Lumpur, Malaysia; 3 MRC Centre for Reproductive Health, Queen’s Medical Research Institute, Edinburgh BioQuarter, Edinburgh, UK; 4 Reproductive Medicine Research Group, School of Medicine, Ninewells Hospital and Medical School, University of Dundee, Dundee, UK

**Keywords:** antioxidants, carnitine, male infertility, reactive oxidative species, sperm

## Abstract

Fifteen percent of couples are globally estimated to be infertile, with up to half of these cases attributed to male infertility. Reactive oxidative species (ROS) are known to damage sperm leading to impaired quantity and quality. Although not routinely assessed, oxidative stress is a common underlying pathology in infertile men. Antioxidants have been shown to improve semen analysis parameters by reducing ROS and facilitating repair of damage caused by oxidative stress, but it remains unclear whether they improve fertility. Carnitines are naturally occurring antioxidants in mammals and are normally abundant in the epididymal luminal fluid of men. We conducted a systematic review and meta-analysis to evaluate the safety and efficacy of carnitine supplementation for idiopathic male infertility. We searched ClinicalKey, ClinicalTrials.gov, Cochrane Central Register of Controlled Trials (CENTRAL), EMBASE, MEDLINE, PubMed and ScienceDirect for relevant studies published from 1 January 2000 to 30 April 2020. Of the articles retrieved, only eight randomised controlled trials were identified and included. Analysis showed that carnitines significantly improve total sperm motility, progressive sperm motility and sperm morphology, but without effect on sperm concentration. There was no demonstrable effect on clinical pregnancy rate in the five studies that included that outcome, although patient numbers were limited. Therefore, the use of carnitines in male infertility appears to improve some sperm parameters but without evidence of an increase in the chance of natural conception.

**Lay summary:**

Although male infertility affects 1:15 men, there is no obvious reason in the vast majority of cases. Reactive oxidative species (ROS) are highly active molecules containing oxygen and are natural byproducts of normal metabolism. However, high concentrations of ROS have been shown to damage sperm, which negatively impacts a couple’s ability to conceive. Carnitines are natural antioxidants found in the body that counterbalance the damaging effects of ROS. We conducted a comprehensive review of published studies to assess whether carnitine supplements are safe and effective in improving sperm quality and pregnancy rates. Our analysis shows that carnitines improve sperm swimming and production of normal-shaped sperm cells but do not affect sperm count or pregnancy rates, although there are only a few studies and scientific evidence is limited. Whilst it is possible that carnitines may benefit male infertility, more evidence is required regarding chances of pregnancy after carnitine therapy.

## Introduction

Infertility is the inability to conceive naturally within 1 year for a sexually active couple not using contraception (Rowe *et al.* 2000, World Health Organization 2020). Worldwide, 15% of couples are estimated to be infertile and approximately 50% of these cases are due to male factor, either as the sole underlying cause or a contributory factor (Agarwal *et al.* 2015). Diagnosis of male infertility usually follows semen analysis. The results may show abnormal semen parameters such as oligozoospermia, asthenozoospermia and teratozoospermia or a combination of these, or a complete absence of sperm in the ejaculate (azoospermia), which is identified in 10–15% of infertile men (Rowe *et al.* 2000, Gudeloglu & Parekattil 2013, Colpi *et al.* 2018). Notably, up to 75% of male infertility is thought to be idiopathic (i.e. with no cause identified) (Punab *et al.* 2017).

Oxidative stress (OS) occurs when there is an overproduction of oxidative free radicals and ROS, which damage spermatozoa and cause male infertility by impairing both the structure and function of sperm (Aitken *et al.* 2003, Aitken & Baker 2006, Valko *et al.* 2007, Venkatesh *et al.* 2011, Agarwal *et al.* 2014). Although the exact mechanism(s) of OS in reducing sperm quality is unknown, it is widely acknowledged that depleted intracellular ATP levels, insufficient axoneme phosphorylation and lipid peroxidation of the cell membrane manifests as poor motility and sperm dysfunction, including reduced ability of sperm to fertilise the oocyte (Storey 1997, Gomez *et al.* 1998, Valko *et al.* 2007). Sperm are vulnerable to OS as they have minimal cytoplasm and endogenous antioxidant protection (Martins da Silva 2019). This leads to the production of malondialdehyde (MDA) and 4-hydroxynonenal (4HNE), which oxidises the lipid membrane and causes fragmentation of both nuclear and mitochondrial DNA in sperm (de Lamirande & Gagnon 1992, Kodama *et al.* 1997, Gomez *et al.* 1998, Aitken *et al.* 2012, Iommiello *et al.* 2015).

There are currently no clinically established treatments available for unexplained male infertility (Martins da Silva *et al.* 2017). Empirical medical treatments such as human menopausal gonadotrophin (hMG)/human chorionic gonadotrophin (hCG), androgen, antioestrogens (clomiphene and tamoxifen), prolactin inhibitors (bromocriptine), and steroids have been used. However, beneficial effects on semen parameters are not proven (Isidori *et al.* 2006, Jungwirth *et al.* 2012). Lifestyle modification advice such as smoking, alcohol cessation and weight reduction programmes are therefore the mainstay of managing male infertility, before progressing to assisted reproduction. Vitamin and dietary supplements are widely marketed to improve male reproductive health and have gained considerable popularity in recent years. However, many formulations are not evidence based (Martins da Silva 2019).

Carnitines are naturally occurring compounds in mammals (Bremer 1983, Reuter & Evans 2012). Primary sources of carnitines are through dietary intake, *de novo* biosynthesis, and renal tubular reabsorption (Reuter & Evans 2012). Foods rich in carnitines include red meats, fish, poultry and dairy products (Steiber *et al.* 2004). Aside from dietary consumption, approximately 25% of total body carnitine is synthesised by the body from the essential amino acids lysine and methionine (Vaz & Wanders 2002, Shekhawat *et al.* 2013). Endogenous plasma and tissue concentrations of carnitines are preserved at relatively precise limits to facilitate mitochondrial and peroxisomal fatty acid oxidation (Bremer 1983, Reuter & Evans 2012). L-carnitine facilitates the β-oxidation of long-chain fatty acids, and in its active form of L-acetylcarnitine, is a vital antioxidant that protects the sperm mitochondria from oxidative stress (Kerner & Hoppel 1998, Russo *et al.* 2000, Abdelrazik *et al.* 2009). Carnitines participate in the metabolism of branch-chain amino acids and stabilise cellular membranes (Shalev *et al.* 1986, Adeva-Andany *et al.* 2017) and can also act as free radicle scavengers, thereby increasing antioxidative capabilities in spermatozoa resulting in reduction of OS (Balercia *et al.* 2005, Dokmeci 2005, Adewoyin *et al.* 2017). *In vitro*, addition of carnitine to culture media increases sperm motility and vitality (Tanphaichitr 1977, Banihani *et al.* 2014). Notably, men with abnormal semen parameters have been reported to have significantly lower carnitine serum levels (Zopfgen *et al.* 2000, Mongioi *et al.* 2016). In this review we have aggregated and analysed currently available data from clinical trials of L-carnitine and/or L-acetylcarnitine in idiopathic male infertility to determine whether carnitine supplements indeed improve sperm quality, and therefore male reproductive potential, in couples with male factor infertility (Haje & Naoom 2015, Moolenaar *et al.* 2015).

## Materials and methods

Our study is based on the Preferred Reporting Items for Systematic Reviews and Meta-Analyses Protocols (PRISMA-P) and the systematic review is reported according to PRISMA guidelines (Moher *et al.* 2015, Shamseer *et al.* 2015). The study protocol is registered in PROSPERO (CRD42020181104).

### Eligibility criteria

Analysis specifically included only Randomised Controlled Trials (RCTs). The RCTs had to be human studies with male patients between the ages of 18 and 65, with abnormal semen characteristics according to WHO normative ranges (2010) and treated with L-carnitine and/or L-acetyl-carnitine (World Health Organization 2010). Studies required at least one control group treated with placebo or without treatment.

Reviews, commentaries, observational studies, retrospective studies, quasi-randomised trials, case series and case reports were excluded. We also excluded literature with animal studies, laboratory and *in vitro* studies, female factor infertility, undiagnosed patients, infertility <1 year, couples with no regular sexual intercourse and other causes of male infertility not related to abnormal semen analysis.

### Information sources

Literature search strategies were developed using medical subject heading (MeSH) terms and text relating to the impact of L-carnitine and L-acetylcarnitine on male reproductive potential. We searched ClinicalKey, ClinicalTrials.gov, Cochrane Central Register of Controlled Trials (CENTRAL), EMBASE, MEDLINE, PubMed and ScienceDirect thoroughly according to PRISMA guidelines (Moher *et al.* 2015, Shamseer *et al.* 2015). The literature search was limited to the English language and published between 1 January 2000 and 30 April 2020. Articles were also sourced by screening through the references of included studies or relevant reviews during the selection process.

### Search strategy

Our MeSH terms were ‘male infertility’ or ‘male reproductive potential’ or ‘male subfertility’ or ‘spermatozoa’ or ‘asthenozoospermia’ or ‘oligospermia’ or ‘oligoasthenozoospermia’ or ‘teratozoospermia’ or ‘DNA damage’ or ‘oxidative stress and ‘Carnitine’ or ‘Levocarnitine’ or ‘L-carnitine’ or ‘L-acetylcarnitine’ or ‘L-acetyl Carnitine’ or ‘L-acetyl-carnitine’ or ‘L-acetyl Carnitine’ or ‘Levoacetylcarnitine’ or ‘Levo-acetyl-carnitine’ or ‘Levoacetyl Carnitine’ or ‘Levo-acetyl Carnitine’ or ‘Acetyl-L-carnitine’ or ‘Acetyl L-carnitine’ or ‘Acetyl-L Carnitine’ or ‘Acetyl-Levocarnitine’ or ‘Acetyl Carnitine’.

### Data management and collection

Covidence was used to filter duplicates and conduct the systematic review data collection process (Veritas Health Innovation). The selection was performed according to PRISMA guidelines (Moher *et al.* 2015, Shamseer *et al.* 2015). All available pieces of literature were thoroughly screened using the inclusion and exclusion criteria through Covidence (Veritas Health Innovation). Literature was first screened by title and abstract. Full-text articles that fulfilled the inclusion criteria were then reviewed. If two or more reports had repeated data, the study with the largest sample size, most extended follow-up, and most specific intervention and outcomes were selected. The screening and selection process was carried out by two independent review authors simultaneously (Khaw and Wong) using a standardised form to include study characteristics such as methodology, number of participants, demographics of participants, detailed test and control interventions, primary and secondary outcomes of the studies, the effect of treatment and risk of bias. Missing data were requested from study authors. Any discrepancies were resolved through consensus.

### Risk of bias in individual studies

The Cochrane Collaboration’s tool for assessing the risk of bias in randomised trials was used to determine the six domains of bias (Higgins *et al.* 2011). Two independent review authors conducted this assessment (Khaw and Wong) and any discrepancies were resolved through consensus. Articles were assessed for bias based on the following aspects: random sequence generation, allocation concealment, blinding of participants and personnel, blinding of outcome assessment, incomplete outcome data, selective reporting and other bias (Higgins *et al.* 2011). Each aspect was then classified as high, low, or unclear risk of material bias (Higgins *et al.* 2011). The risk of bias assessment chart was then generated using Review Manager (RevMan) 5.4 software (The Nordic Cochrane Centre 2014).

### Data synthesis

We then carried out a descriptive analysis of included studies focusing on the methodology of the study, type and details of the intervention, target population demographics, primary outcomes, secondary outcomes, adverse outcomes and intervention effects. A meta-analysis was conducted for studies with the same intervention and comparator with equal outcome measures. For the meta-analysis, we conducted a random-effects meta-analysis using risk ratios for dichotomous outcomes and mean differences with s.d. The raw mean differences were used instead of standardised mean differences, as all studies used the same continuous outcomes and units of measure. We then used the statistical significance of 95% CIs and *P*-values for each outcome. Where there were results from multiple durations of therapy, the results after the most prolonged period of treatment was used. Higgins’s I^2^ test statistic (>50% indicative of substantial heterogeneity) was utilised to assess heterogeneity among the studies. Cochran’s Q test was not used to analyse heterogeneity as there were only small numbers of available studies. We then proceed with a stratified meta-analysis for study quality, trial size, concealment of allocation, blind adjudication of events, analysis according to the intention-to-treat principle, and intervention method. Assessment evidence of publication bias was carried out for the included studies and plots were generated to visually inspect the data through a funnel plot generated by RevMan 5.4 software (The Nordic Cochrane Centre 2014). In study outcomes that had substantial heterogeneity, the data were also synthesised through a narrative and qualitative approach.

The s.d. from Sigman *et al.* (2006) was calculated according to the Cochrane Handbook for Systematic Reviews of Interventions section 6.5.2.3(3) (Higgins *et al.* 2019).

### Overall quality of evidence

The quality and consistency of each comparison was assessed using the Grading of Recommendations Assessment, Development and Evaluation (GRADE) guidelines through GRADEpro (Balshem *et al.* 2011, Schünemann *et al.* 2013, Evidence Prime 2015). The strength of evidence for critical and essential outcomes was rated based on study design, risk of bias, consistency, limitations, directness, reporting precision and publication bias (Balshem *et al.* 2011, Schünemann *et al.* 2013, Evidence Prime 2015). Table 1 shows a summary of the included studies and their GRADE assessments. Effect (risk) of carnitine is expressed as mean difference (MD).

**Table 1:** Summary of findings of carnitine compared to placebo or no treatment for idiopathic male infertility.

Outcomes	Anticipated absolute effects (95% CI)			RR (95% CI)	Participants in studies		Certainty of evidence	
	Risk with placebo or no treatment, range	Risk with carnitine						
		Value	Range		*n*	Studies	Evidence	Grade
Sperm concentration	0.8–33.73 million/mL	MD 2.7 million/mL higher	2.04 lower to 7.44 higher	–	438	6 RCTs	⊕⊝⊝⊝	VERY LOW^a,b,c^
Total sperm motility	3.3–43.4%	MD 10.72% higher	3.94 higher to 17.5 higher	–	459	7 RCTs	⊕⊕⊝⊝	LOW^a,c^
Progressive sperm motility	4–24.41%	MD 9.82% higher	2.01 higher to 17.62 higher	–	231	3 RCTs	⊕⊕⊝⊝	LOW^a,c^
Normal sperm morphology	1.39–32.73%	MD 2.41% higher	0.79 higher to 4.03 higher	–	438	6 RCTs	⊕⊕⊝⊝	LOW^a,c^
Clinical pregnancy								
Study population	113 per 1000	116 per 1000	61–221	1.03 (0.54–1.96)	301	5 RCTs	⊕⊕⊝⊝	LOW^a,b^

The population was men with abnormal semen characteristics. The intervention was l-carnitine and/or l-acetylcarnitine. The table compares placebo or no treatment. The outcomes measured were semen analysis parameters; clinical pregnancy; adverse events in a clinic or hospital.

^a^Lack of blinding; ^b^Crosses the line of no effect; ^c^Higgins’s I^2^ test >50%.

## Results

### Study characteristics

**Figure 1:**
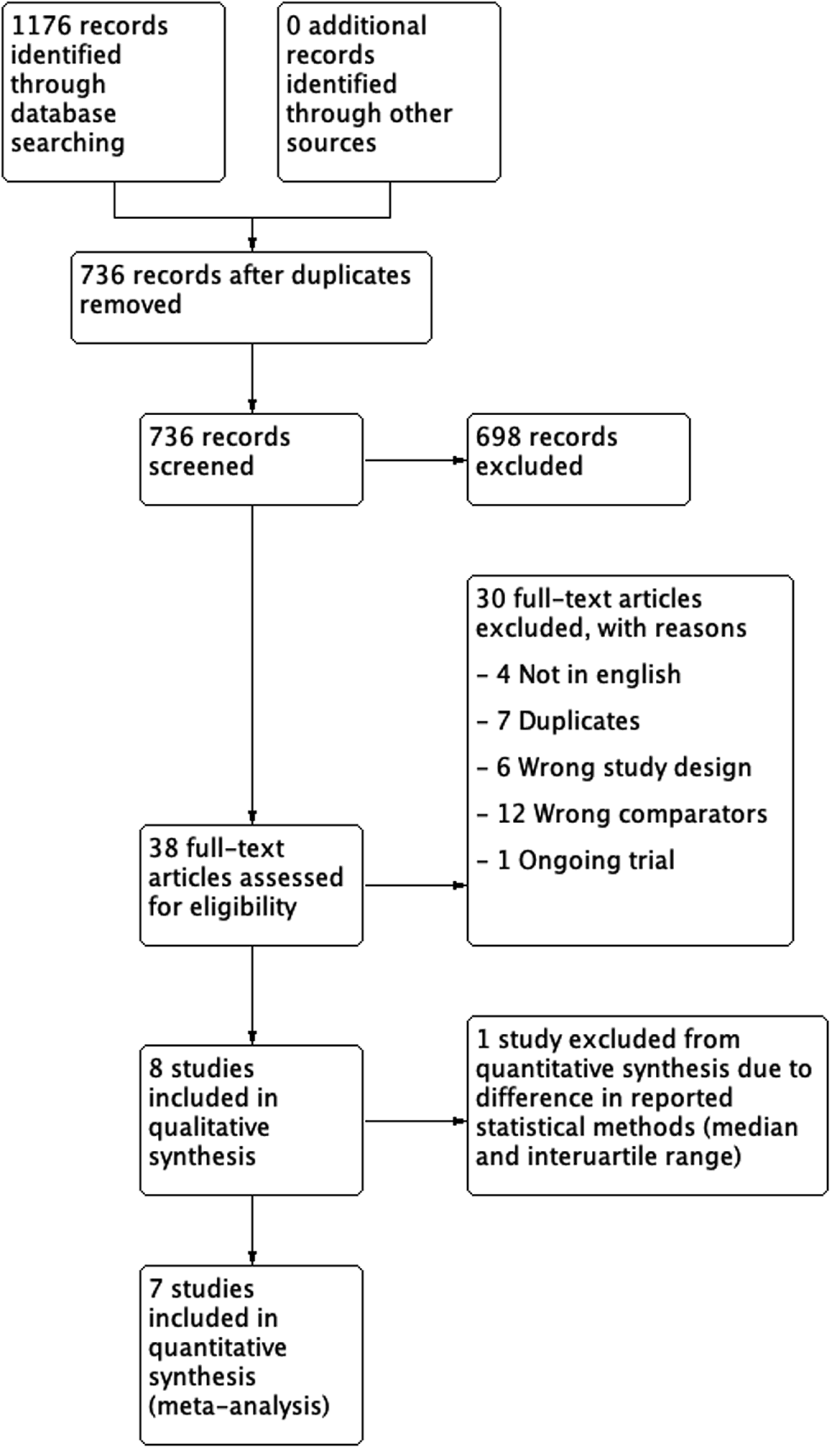
PRISMA flowchart.

The search strategy identified 1176 citations dated 1 January 2000 to 30 April 2020. After 440 duplicates were removed, 736 abstracts were assessed. Six hundred and ninety-eight records were excluded as they did not meet inclusion criteria. Thirty-eight full-text articles were searched for eligibility according to inclusion and exclusion criterion. All included studies were of randomised controlled trials without cross over. We excluded Lenzi 2003 as it had a cross-over design (Lenzi *et al.* 2003). A total of eight studies were included in the review and the findings from seven studies were pooled into a meta-analysis (Cavallini *et al.* 2004, Lenzi *et al.* 2004, Balercia *et al.* 2005, Sigman *et al.* 2006, Dimitriadis *et al.* 2010, Mehni *et al.* 2014, Haje & Naoom 2015, Tsounapi *et al.* 2018). The data by Cavellini *et al.* was excluded from the meta-analysis as the authors reported their results as medians and interquartile ranges rather than means and s.d. (Cavallini *et al.* 2004). Our search process is summarised in the PRISMA flowchart (Fig. 1).

**Figure 2:**
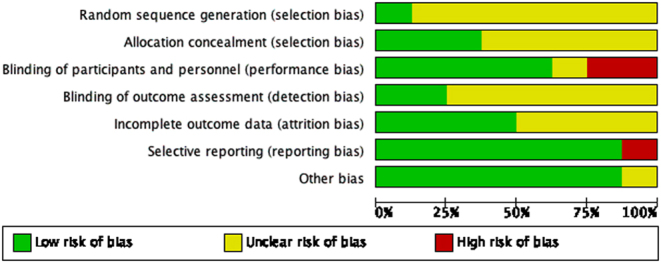
Risk of bias summary.

**Figure 3:**
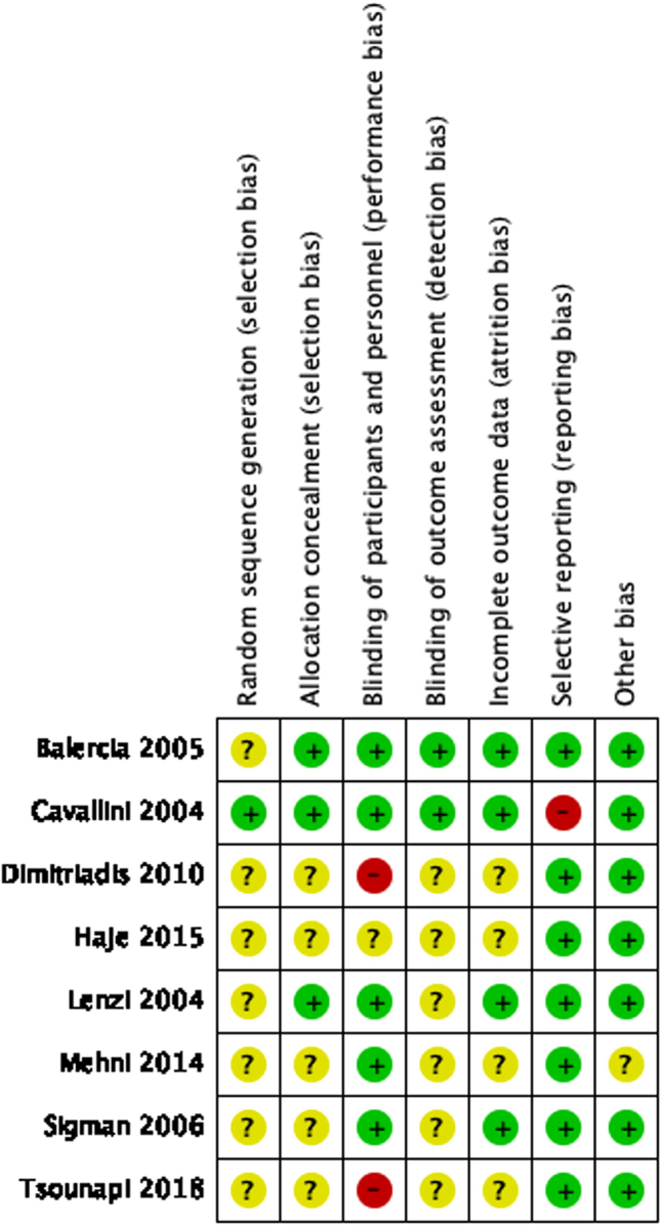
Risk of bias graph.

The included articles were assessed based on seven aspects: random sequence generation, allocation concealment, blinding of participants and personnel, blinding of outcome assessment, incomplete outcome data, selective reporting and other bias (Figs 2 and 3). Where additional information was required, the study authors were contacted but this was unsuccessful. Overall, none of the included studies explicitly mentioned the method of randomisation (Cavallini *et al.* 2004, Lenzi *et al.* 2004, Balercia *et al.* 2005, Sigman *et al.* 2006, Mehni *et al.* 2014, Dimitriadis *et al.* 2010, Haje & Naoom 2015, Tsounapi *et al.* 2018). Hence, all literature had an unclear risk of selection bias in this aspect. The majority of articles also had unclear risks of detection bias as the process of assessment was not reported in detail (Lenzi *et al.* 2004, Sigman *et al.* 2006, Dimitriadis *et al.* 2010, Mehni *et al.* 2014, Haje & Naoom 2015, Tsounapi *et al.* 2018).

One study (Tsounapi *et al.* 2018) was five-armed, four studies (Balercia *et al.* 2005, Dimitriadis *et al.* 2010, Mehni *et al.* 2014, Haje & Naoom 2015) were four-armed, one study (Cavallini *et al.* 2004) was three-armed, and two studies (Lenzi *et al.* 2004, Sigman *et al.* 2006) were two-armed. The total duration of treatment ranged from 3 months (Dimitriadis *et al.* 2010, Mehni *et al.* 2014, Haje & Naoom 2015, Tsounapi *et al.* 2018) to 6 months (Cavallini *et al.* 2004, Lenzi *et al.* 2004, Balercia *et al.* 2005, Haje & Naoom 2015) while follow-up time varied between from 3 months (Mehni *et al.* 2014) to9 months (Cavallini *et al.* 2004, Balercia *et al.* 2005). Table 2 shows an overview of the included studies.

**Table 2:** Study characteristics.

	Study	Study design	Age (years)	Treatment/day	Control	Arms	Duration of treatment (Weeks)	Sample size		Total follow-up time
								Therapy	Control	
2004	Cavallini *et al.* (2004)	RCT	27–40	2 g LC + 1 g LAC	Placebo	3	24	39	47	9 months
2004	Lenzi *et al.* (2004)	RCT	20–40	2 g LC + 1 g LAC	Placebo	2	24	30	26	8 months
2005	Balercia *et al.* (2005)	RCT	24–38	2 g LC + 1 g LAC (*n* = 14) vs 3 g LC (*n* = 15) vs 3 g LAC (*n* = 15)	Placebo	4	24	44	15	9 months
2006	Sigman *et al.* (2006)	RCT	36.2 ± 1.7	2 g LC + 1 g LAC	Placebo	2	16	12	9	6 months
2010	Dimitriadis *et al.* (2010)	RCT	NR	1 g LC	No TT	4	12	26	22	13 weeks (6 days after the experimental period)
2014	Mehni *et* *al.* (2014)	RCT	25–40	1 g LC	Placebo	4	12	51	59	3 months
2015	Haje & Naoom (2015)	RCT	37.54 ± 2.46	1 g LC	Placebo	4	12–24	20	29	4–7 months (as two samples were taken after treatment – 1 month apart)
2018	Tsounapi *et al.* (2018)	RCT	NR	1 g LC	No TT	5	12.8	44	42	Experimental period of 90 days; up to 180 days for pregnancy rate

NR, Not reported; TT, treatment.

### Population

Our meta-analysis only included studies with idiopathic male infertility. The participants were treated with carnitine supplementation, placebo or did not receive any treatment. Six studies (438 men) recorded sperm concentration and morphology, seven studies (459 men) reported total sperm motility and three studies (231 men) evaluated the progressive sperm motility after carnitine therapy. Only four studies (252 men) reported pregnancy outcomes following carnitine supplementation. All participants were between the ages of 18 and 65 with infertility of more than 1 year. A study by Cavallini *et al.* (2004) was also included in our systematic review, but not in the meta-analysis, as their results were recorded in medians and interquartile ranges. Their study also enrolled men with varicocele but only the results from men with idiopathic male infertility were included in our review (Cavallini *et al.* 2004).

### Interventions

Data included in our analysis compared l-carnitine (LC) and/or l-acetylcarnitine (LAC) to placebo or no treatment. Four studies (Cavallini *et al.* 2004, Lenzi *et al.* 2004, Balercia *et al.* 2005, Sigman *et al.* 2006) compared LC and LAC to placebos, five studies compared LC to placebo (Balercia *et al.* 2005, Mehni *et al.* 2014, Haje & Naoom 2015) or no treatment (Dimitriadis *et al.* 2010, Tsounapi *et al.* 2018) while only one study (Balercia *et al.* 2005) compared LAC to placebo.

### Outcomes

The primary outcomes for our review are sperm concentration, total sperm motility, progressive sperm motility, sperm morphology, pregnancy rate and live birth rate. We also included sperm DNA damage and adverse events such as side effects and miscarriage as secondary outcomes. Seven studies (Lenzi *et al.* 2004, Balercia *et al.* 2005, Sigman *et al.* 2006, Dimitriadis *et al.* 2010, Mehni *et al.* 2014, Haje & Naoom 2015, Tsounapi *et al.* 2018) recorded the total sperm motility while only four studies (Cavallini *et al.* 2004, Lenzi *et al.* 2004, Balercia *et al.* 2005, Tsounapi *et al.* 2018) reported progressive sperm motility. Sperm concentration and morphology were recorded by seven studies (Cavallini *et al.* 2004, Lenzi *et al.* 2004, Balercia *et al.* 2005, Dimitriadis *et al.* 2010, Mehni *et al.* 2014, Haje & Naoom 2015, Tsounapi *et al.* 2018). None of the studies included DNA damage assessment. Although five studies (Cavallini *et al.* 2004, Balercia *et al.* 2005, Sigman *et al.* 2006, Haje & Naoom 2015, Tsounapi *et al.* 2018) reported on pregnancy rate, none included live birth or miscarriage data. We contacted the authors where details were unclear or if a different statistical approach was used in their study; however, we received no response.

### Sperm concentration

**Figure 4:**
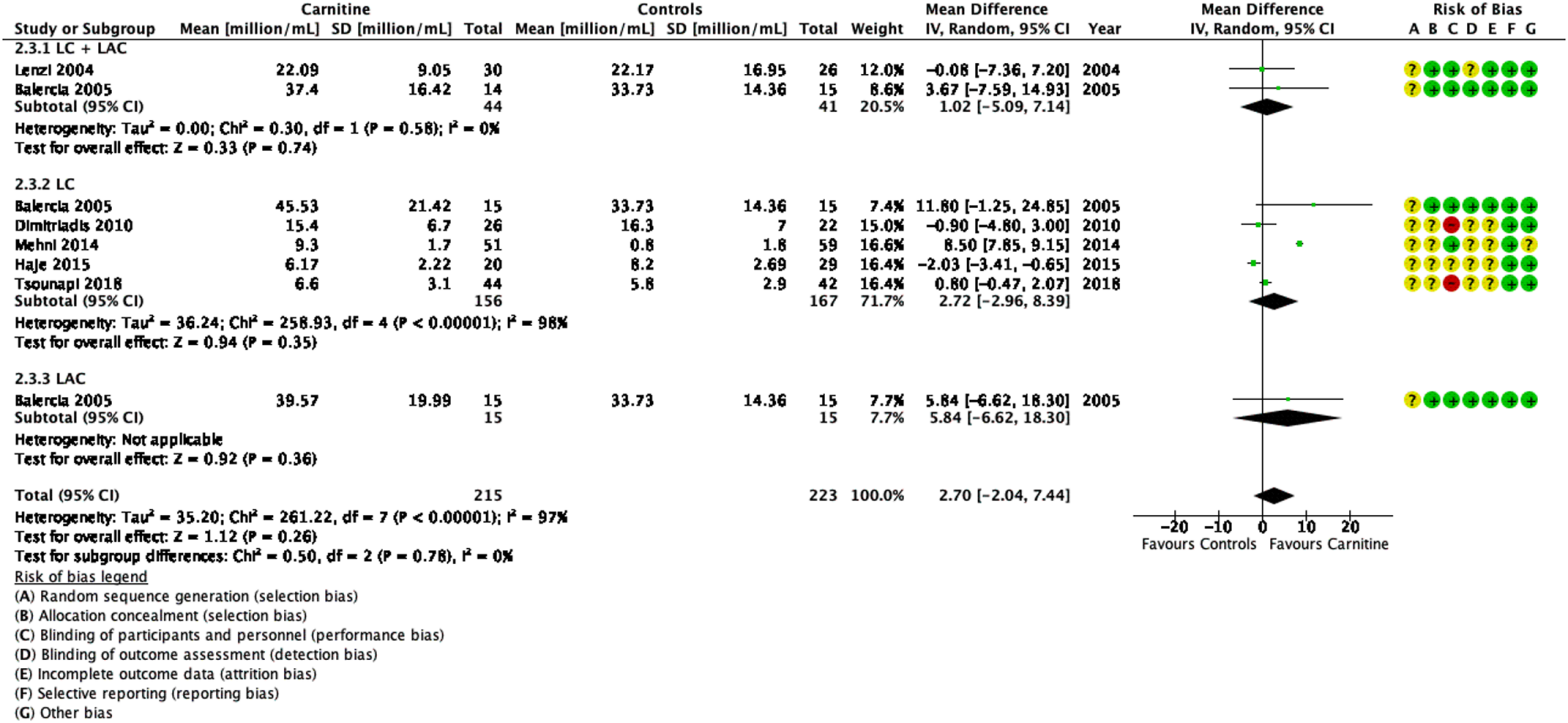
Forest plot of comparison for sperm concentration.

Seven studies reported the effects of carnitines on sperm concentration (Fig. 4) (Cavallini *et al.* 2004, Lenzi *et al.* 2004, Balercia *et al.* 2005, Dimitriadis *et al.* 2010, Mehni *et al.* 2014, Haje & Naoom 2015, Tsounapi *et al.* 2018). Although the study by Cavallini *et al.* reported higher sperm concentrations after LC + LAC therapy (20.6%, IQR 24.9–15.1%) when compared to a placebo (10.9%, IQR 15.1–9.0%), findings were reported as median and interquartile range rather than mean and s.d. and no statistical analysis was reported (Cavallini *et al.* 2004). This study was therefore excluded from the meta-analysis.

Overall, our findings showed that carnitines did not significantly improve sperm concentration (*P* > 0.05). However, the six studies showed a high heterogeneity (MD 2.70, 95% CI −2.04 to 7.44; *n* = 438, RCT = 6, *P* > 0.05, I^2^ = 97%) (Lenzi *et al.* 2004, Balercia *et al.* 2005, Dimitriadis *et al.* 2010, Mehni *et al.* 2014, Haje & Naoom 2015, Tsounapi *et al.* 2018) primarily due to two studies with very small s.d. compared to the others (Mehni *et al.* 2014, Haje & Naoom 2015). A sensitivity analysis after removal of these two studies showed homogeneity between the remaining studies but the meta-analysis still showed no significant effects of carnitines on sperm concentration (MD 0.79, 95% CI −0.39 to 1.96; *n* = 279, RCT = 4, *P* > 0.05, I^2^ = 0%) (Lenzi *et al.* 2004, Balercia *et al.* 2005, Dimitriadis *et al.* 2010, Tsounapi *et al.* 2018).

### Total sperm motility

**Figure 5:**
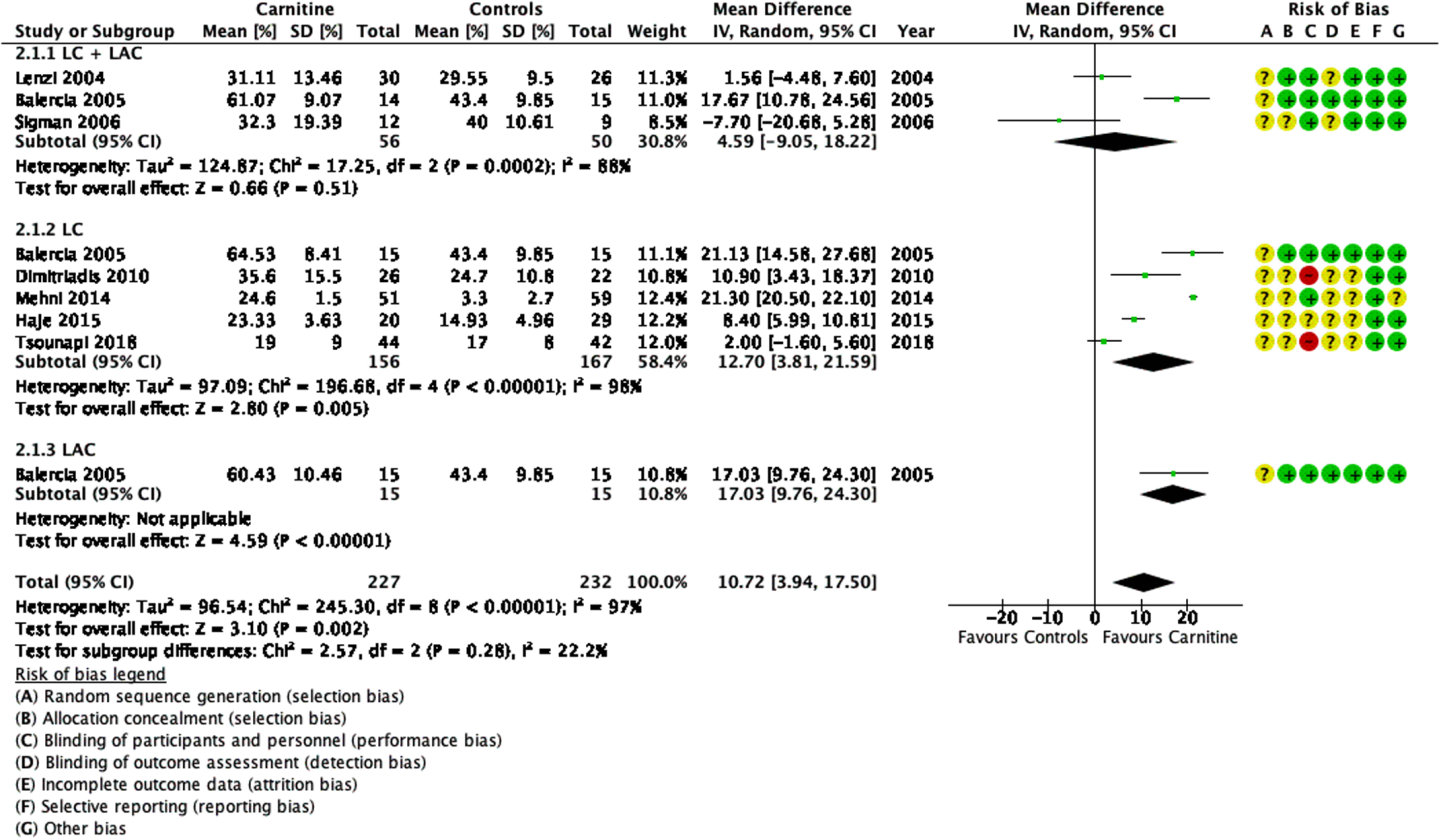
Forest plot of comparison for total sperm motility.

Seven studies compared the efficacy of carnitines to placebo or no treatment (Lenzi *et al.* 2004, Balercia *et al.* 2005, Sigman *et al.* 2006, Dimitriadis *et al.* 2010, Mehni *et al.* 2014, Haje & Naoom 2015, Tsounapi *et al.* 2018). Analysis of the mean difference in total sperm motility showed that carnitines improved total sperm motility by 10.72% (95% CI 3.94–17.50; *n* = 459, RCT = 7, *P* < 0.05, I^2^ = 97%) (Fig. 5).

The studies showed high heterogeneity. In studies comparing LC and LAC to placebo, Balercia *et al.* showed a significant increase in total sperm motility in all three study arms when compared to a placebo (MD 18.75, 95% CI 14.78–22.73; *n* = 30, *P* < 0.05) (Balercia *et al.* 2005). However two other studies did not show significant differences between the treatment and control groups (Lenzi *et al.* 2004, Sigman *et al.* 2006). Lenzi *et al.* showed a mean difference of 1.56 (95% CI −4.48 to 7.60; *n* = 56, *P* > 0.05), while Sigman *et al.* showed a mean difference of −7.70 (95% CI −20.68 to 5.28; *n* = 21, *P* > 0.05). In studies that compared LC to placebo or no treatment, four studies (Balercia *et al.* 2005, Dimitriadis *et al.* 2010, Mehni *et al.* 2014, Haje & Naoom 2015) showed significant improvements after receiving LC while one study (Tsounapi *et al.* 2018) reported no significant differences. Balercia *et al.* (2005) was the only study that assessed LAC treatment alone.

A detailed statistical analysis is shown in Fig. 5.

### Progressive sperm motility

**Figure 6:**
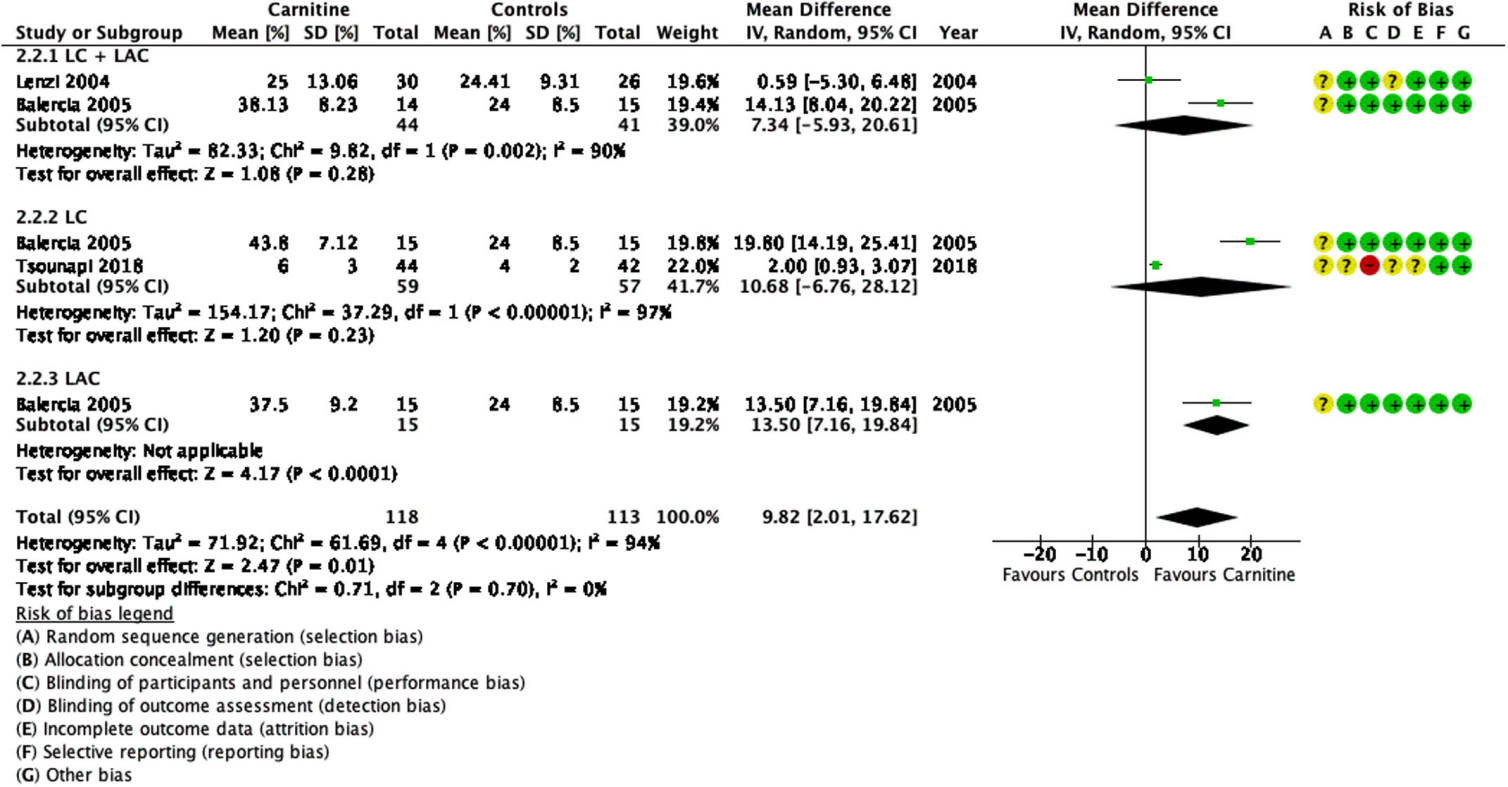
Forest plot of comparison for progressive sperm motility.

Four studies showed an increase in progressive sperm motility after carnitine when compared to control groups (Cavallini *et al.* 2004, Lenzi *et al.* 2004, Balercia *et al.* 2005, Tsounapi *et al.* 2018). Overall, carnitines significantly improved progressive sperm motility in idiopathic male infertility (MD 9.82, 95% CI 2.01, 17.62; *n* = 231, *P* < 0.05) (Fig. 6).

This outcome also showed high heterogeneity (I^2^ = 94%). Balercia *et al.* (2005) showed significant improvement in progressive sperm motility in all LC, LAC and LC + LAC therapy groups (MD 16.02, 95% CI 11.98–20.06; *n* = 30, *P* < 0.05). Tousnapi *et al.* (2018) reported a significant increase in progressive sperm motility after LC therapy (MD 2.00, 95% CI 0.93–3.07; *n* = 86, *P* < 0.05). Lenzi *et al.* (2004) did not show a significant increase in progressive sperm motility after LC + LAC therapy (MD 0.59, 95% CI −5.30 to 6.48; *n* = 56, *P* > 0.05). Cavallini *et al.* (2004) reported an increase in progressive sperm motility after LC + LAC therapy (23.6%, IQR 28.9–16.0%) when compared to controls (13.2%, 18.6–9.0%) but no raw data or *P*-values were provided to draw a statistically significant conclusion.

### Sperm morphology

**Figure 7:**
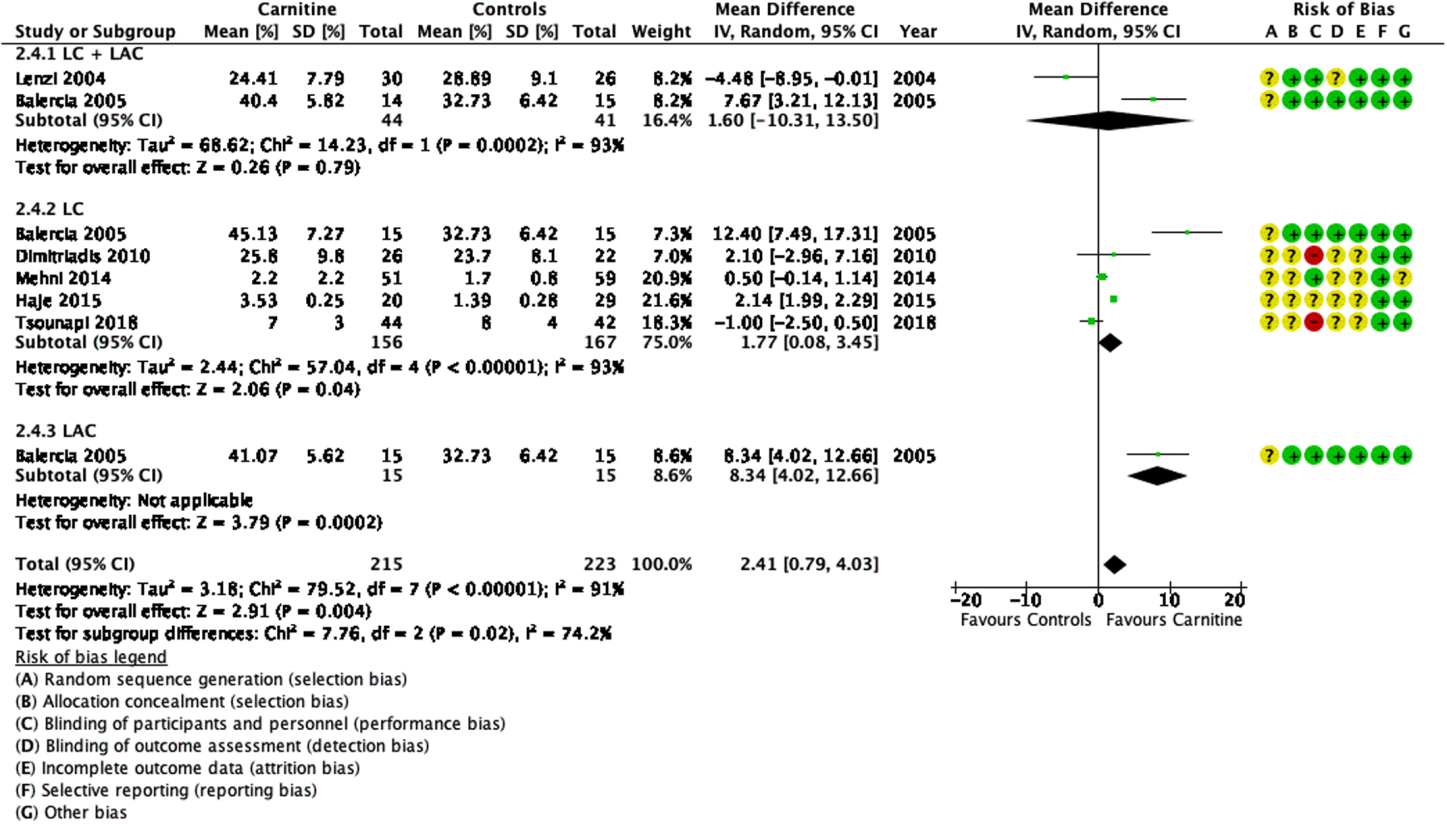
Forest plot of comparison for normal sperm morphology.

The results of sperm morphology were recorded by seven studies (Cavallini *et al.* 2004, Lenzi *et al.* 2004, Balercia *et al.* 2005, Dimitriadis *et al.* 2010, Mehni *et al.* 2014, Haje & Naoom 2015, Tsounapi *et al.* 2018). Overall, our results showed a significant improvement in sperm morphology (*P* < 0.05) as seen in Fig. 7.

This outcome also had a high heterogeneity (I^2^ = 91%). Balercia *et al.* (2005) documented a significant improvement of 9.29% when compared to a placebo (MD 9.29, 95% CI 6.51–12.06; *n* = 30, *P* < 0.05). Lenzi *et al.* (2004) showed no significant differences between the treatment and control groups after LC + LAC therapy. Among the remaining studies of treatment after LC, only the study by Haje and Naoom (2015) showed a significant improvement (MD 2.14, 95% CI 1.99–2.29; *n* = 49, *P* < 0.05) in sperm morphology; while three other studies (Dimitriadis *et al.* 2010, Mehni *et al.* 2014, Tsounapi *et al.* 2018) recorded no significant changes. Cavallini *et al.* (2004) recorded an improvement of sperm morphology in their study after LC + LAC therapy (27.3%, IQR 32.0–22.6% vs 15.3%, IQR 22.0–12.1% in the placebo group) but the data provided is not sufficient for a test of statistical significance.

### Clinical pregnancy rate

**Figure 8:**
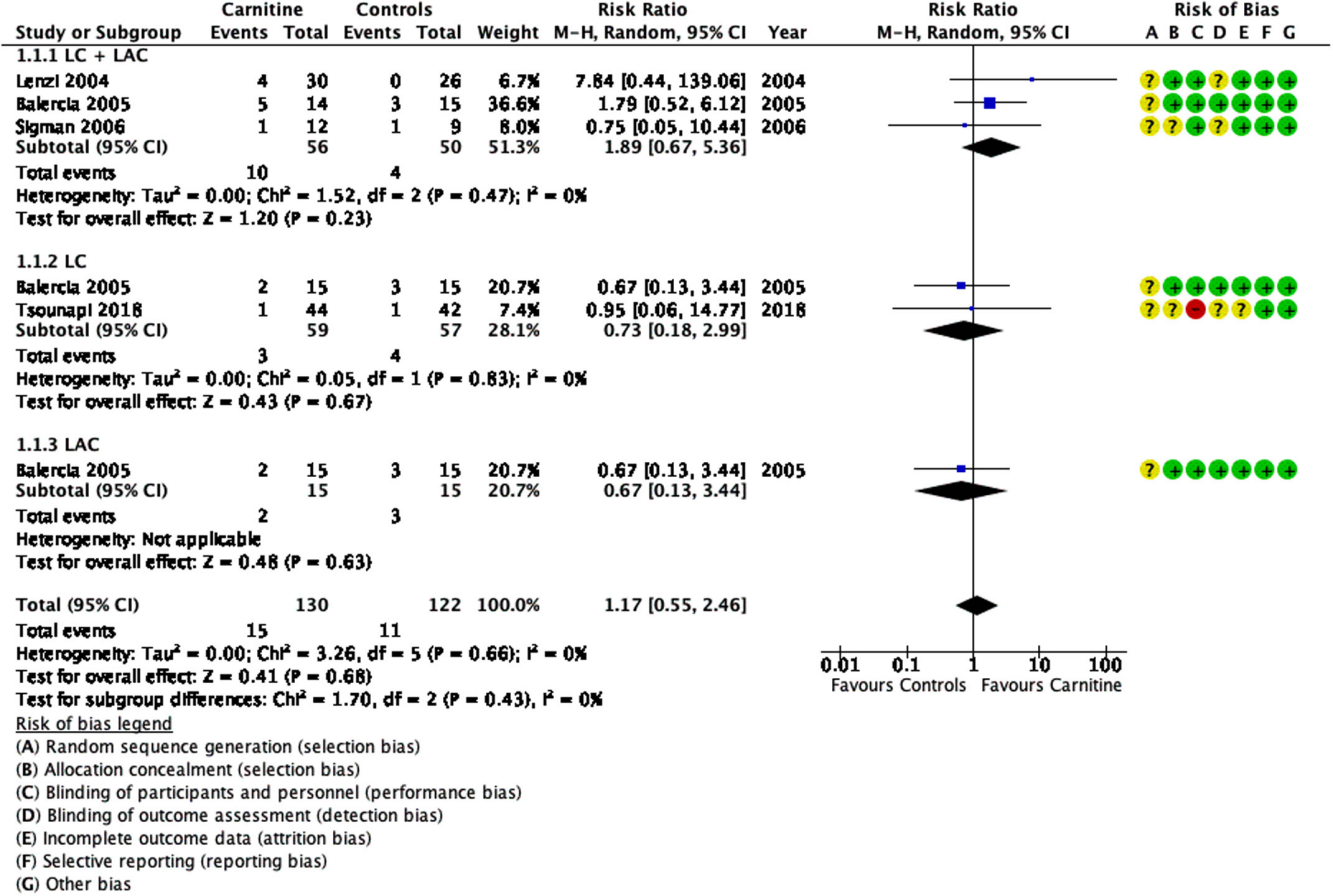
Forest plot of comparison for clinical pregnancy.

A total of five RCTs were analysed for their reported pregnancies and four studies were consolidated into a meta-analysis. There was low heterogeneity (I^2^ = 0%) and some concern with the risk of bias. The meta-analysis (Fig. 8) showed that there was no significant improvement in pregnancy rates when compared to control groups (RR 1.17, 95% CI 0.55–2.46; *n* = 252, RCT = 4, *P* > 0.05).

In patients treated with a combination of LC and LAC, Cavallini *et al.* (2004) showed a significant improvement in pregnancy rate (X^2^ = 20.795, *P* < 0.01) when compared to controls. In contrast, the three other studies (Lenzi *et al.* 2004, Balercia *et al.* 2005, Sigman *et al.* 2006) did not show significant differences between the two groups (RR 1.89, 95% CI 0.67–5.36; *n* = 106, RCT = 3, *P* > 0.05, I^2^ = 0%). Patients treated with either LC (RR 0.73, 95% CI 0.28–1.87; *n* = 165, RCT = 3, *P* > 0.05, I^2^ = 0%) or LAC (RR 0.67, 95% CI 0.13–3.44; *n* = 30, RCT = 1, *P* > 0.05) did not show any significant changes in pregnancy rates in comparison to their control groups (Balercia *et al.* 2005, Haje & Naoom 2015, Tsounapi *et al.* 2018). Haje and Naoom (2015) studied the effect of carnitine supplementation in patients undergoing ICSI and reported no significant increase in pregnancy rates.

Overall, the results were imprecise as there were very few events and thus confidence intervals were wide. Moreover, the quality of evidence is also low.

### Adverse events

Six studies did not report adverse events (Lenzi *et al.* 2004, Balercia *et al.* 2005, Dimitriadis *et al.* 2010, Mehni *et al.* 2014, Haje & Naoom 2015, Tsounapi *et al.* 2018). Sigman *et al.* (2006) confirmed that there were no adverse events in their study. Cavallini *et al.* (2004) reported four cases of mild euphoria (two in the LC + LAC group and two in control groups). Their study also recorded two cases of gastrointestinal side effects (mild epigastria and nausea) from both the treatment and control groups. However, these side effects were reported as negligible as they did not result in therapy suspension. None of the studies included data related to miscarriage.

### Carnitine versus other arms in the included studies

Further studies were identified that compared carnitine to other compounds, rather than placebo or no treatment. They were therefore not included in the meta-analysis but are summarised in Table 3.

**Table 3:** Carnitine versus other arms in the included studies.

Published year	Study	Age (years)	Treatment/day	Improved Outcome				
				Sperm concentration, (×10^6^/mL)	Total sperm motility (%)	Progressive sperm motility (%)	Sperm morphology (%)	Clinical pregnancy rate (compared to carnitines or controls)
2004	Cavallini *et al.* (2004)	27–40	LC + LAC and cinnoxicam	Improved*	Unchanged*	Improved*	Improved*	x^2^ = +5.743; *P* < 0.05
2010	Dimitriadis *et al.* (2010)	NR	Vardenafil	+12.0, *P* < 0.05	+19.9, *P* < 0.05	NR	+16.3, *P* < 0.05	NR
			Sildenafil	+14.8, *P* < 0.05	+21.4, *P* < 0.05	NR	+17.7, *P* < 0.05	
2014	Mehni *et al.* (2014)	25–40	LC and Pentoxifylline	*P* = 0.001^$^	*P* = 0.045^$^	NR	*P* = 0.052^$^	NR
2015	Haje & Naoom (2015)	37.54 ± 2.46	Tamoxifen	+3.23, *P* = 0.016	*P* > 0.05	NR	+0.56, *P* = 0.25	48.9%, *P* > 0.05
			Tamoxifen and carnitine	+0.6, *P* = 0.01	+5.75, *P* = 0.045	NR	+1.11, *P* = 0.026	48.3%, *P* > 0.05
2018	Tsounapi *et al.* (2018)	NR	Profertil	+2.1, *P* > 0.05	+16, *P* < 0.05	+9, *P* < 0.05	+2, *P* > 0.05	NR
			Avanafil	+3.5, *P* > 0.05	+30, *P* < 0.05	+12, *P* < 0.05	*P* > 0.05	
			Combination of Profertil and Avanafil	+2.9, *P* > 0.05	+24, *P* < 0.05	+7, *P* < 0.05	+2, *P* > 0.05	

*Detailed statistical data was not reported by the authors, ^$^The raw data was not provided by the authors.

NR, Not reported.

## Discussion

Several published studies have reported that carnitines have beneficial effects on improving sperm quality in men with idiopathic male infertility (Steiber *et al.* 2004, Isidori *et al.* 2005, Isidori *et al.* 2006, Mongioi *et al.* 2016, Smits *et al.* 2019). Notably, concentrations of carnitine have also been documented to be higher in the sperm and seminal plasma of fertile men, compared to men with abnormal semen parameters (Zopfgen *et al.* 2000, Banihani *et al.* 2014, Mongioi *et al.* 2016, Smits *et al.* 2019). The scientific rationale behind this is a vital role played by carnitines during spermatogenesis (Jeulin & Lewin 1996, Agarwal & Sekhon 2011, Aliabadi *et al.* 2012). Carnitines are concentrated in the epididymal luminal fluid (Jeulin & Lewin 1996), and likely to be associated with sperm maturation. Carnitines also scavange free oxygen radicles and ROS, thus protecting against OS, as well as aiding cellular repair in mitochondria during β-oxidation of long-chain fatty acids (Fritz 1963, Steiber *et al.* 2004, Reuter & Evans 2012, Smits *et al.* 2019). However, whilst improved semen characterisitics have been reported, very few studies have recorded pregnancy outcomes after treatment of infertile men with carnitines, and none have considered live birth as a primary outcome (Zini *et al.* 1993, Shekarriz *et al.* 1995, Hakonsen *et al.* 2011, Poljsak 2011, Moolenaar *et al.* 2015).

This meta-analysis presents evidence supporting the improvement of sperm parameters with carnitine supplementation. Carnitines significantly improve total sperm motility (+10.72%), progressive sperm motility (+9.82%) and sperm morphology (+2.41%) (Cavallini *et al.* 2004, Lenzi *et al.* 2004, Balercia *et al.* 2005, Sigman *et al.* 2006, Dimitriadis *et al.* 2010, Mehni *et al.* 2014, Haje & Naoom 2015, Tsounapi *et al.* 2018). There does not appear to be a positive effect of carnitine supplementation on sperm concentration (Cavallini *et al.* 2004, Lenzi *et al.* 2004, Balercia *et al.* 2005, Dimitriadis *et al.* 2010, Mehni *et al.* 2014, Haje & Naoom 2015, Tsounapi *et al.* 2018). However, it is notable that the studies are characterised by high heterogeneity, and the quality of the evidence was low (total sperm motility, progressive sperm motility and sperm morphology) or very low (sperm concentration) when assessed through GRADEpro (Table 1).

The data indicate that carnitine does not significantly improve pregnancy rates in infertile couples with male infertility, despite improvements in sperm motility and morphology. However, natural conception was not a primary outcome in most studies, indeed most did not follow-up until pregnancy. Therefore, more evidence is required to study the effects of carnitines on pregnancy outcomes. Although multiple attempts have been made to encourage RCTs to report on fertility outcomes, very few RCTs achieve this in studies relating to male infertility (Tournaye, 2006). Nonetheless, two recently reported large RCTs showed that folic acid and zinc supplements (FAZST) or combination antioxidant treatment including Vitamin C, Vitamin E, folic acid, selenium, zinc, and l-carnitine (MOXI trial) did not improve clinical pregnancy or live birth rates when compared to placebo (Schisterman *et al.* 2020, Steiner *et al.* 2020).

Our findings are consistent with previously published systematic reviews researching the efficacy and safety of antioxidants in idiopathic male infertility. Two systematic reviews of empirical dietary and/or supplementary intervention recorded improved total sperm motility, progressive sperm motility and sperm morphology (Salas-Huetos *et al.* 2018, Omar *et al.* 2019). However, it is notable that our findings of effects on total sperm motility and morphology differ from a recent meta-analysis of carnitines in men with idiopathic oligoasthenoteratozoospermia conducted by Zhang *et al.* (2020). Their systematic review included studies of carnitine plus other antioxidants/compounds and one study that used active controls (Zhang *et al.* 2020). In contrast, we selected studies of carnitine-only treatment vs non-active controls so these studies were excluded during our full-text screening. We also included additional studies from other database searches (Zhang *et al.* 2020). However, the other authors similarly commented on inconsistent data and high heterogeneity amongst the published trials.

A major limitation of this systematic review, and others, is the inability to assess robustly the effect of carnitines on natural conception and pregnancy outcomes as this has not been comprehensively studied to date. Critically, our findings in regards to pregnancy rates did not support carnitine supplementation as an intervention for male infertility, which disagrees with Zhang *et al.* and is reflective of the different study data included (Zhang *et al.* 2020).

## Conclusion

Overall, our systematic review shows that carnitine supplementation can improve sperm motility and morphology. However, there were only eight randomised controlled trials that specifically compared carnitine(s) to placebo or no treatment and study outcomes had high heterogeneity and were derived from low-quality evidence (Table 1). The majority of studies included found that carnitines were most effective in men with severe idiopathic infertility (Cavallini *et al.* 2004, Balercia *et al.* 2005, Sigman *et al.* 2006, Mehni *et al.* 2014), supporting their use as a potential treatment. However, whilst it is accepted that gains in male fertility are likely to be seen with improvement in total motile count, particularly when at the lower end of the range (Hamilton *et al.* 2015), studies included in this meta-analysis have not demonstrated increase in chance of conception, pregnancy and live birth with carnitine supplementation. It therefore remains unclear whether carnitines are a suitable intervention for idiopathic male infertility and randomised placebo‐controlled trials reporting on pregnancy and live births are required to clarify this.

## Declaration of interest

S Martins da Silva has received research funding from AstraZeneca. The other authors declare no conflict of interests. S Martins da Silva is an Associate Editor of Reproduction and Fertility. S Martins da Silva was not involved in the review or editorial process for this paper, on which she is listed as an author.

## Author contribution statement

S C K conceived and designed the study. S C K and Z Z W performed the acquision of data and quality assessment of included studies. S C K conducted the meta-analysis. S C K, R A A and S M d S analysed and interpreted the data. S C K wrote the manuscript with support and input from R A A and S M d S. R A A and S M d S revised the manuscript critically for important intellectual content. All authors approved the final version of the manuscript to be published.
